# An International Study of Factors Affecting Variability of Dosimetry Calculations, Part 2: Overall Variabilities in Absorbed Dose

**DOI:** 10.2967/jnumed.122.265094

**Published:** 2023-07

**Authors:** Julia Brosch-Lenz, Suqi Ke, Hao Wang, Eric Frey, Yuni K. Dewaraja, John Sunderland, Carlos Uribe

**Affiliations:** 1 Department of Integrative Oncology, BC Cancer Research Institute, Vancouver, British Columbia, Canada;; 2 Division of Quantitative Sciences, Sidney Kimmel Comprehensive Cancer Center, Johns Hopkins University School of Medicine, Baltimore, Maryland;; 3 Rapid, LLC, Baltimore, Maryland;; 4 Department of Radiology, Johns Hopkins University, Baltimore, Maryland;; 5 Department of Radiology, University of Michigan, Ann Arbor, Michigan;; 6 Department of Radiology, University of Iowa, Iowa City, Iowa;; 7 Department of Functional Imaging, BC Cancer, Vancouver, British Columbia, Canada; and; 8 Department of Radiology, University of British Columbia, Vancouver, British Columbia, Canada

**Keywords:** radiopharmaceutical therapy, dosimetry, variability, standardization, dosimetry challenge

## Abstract

Dosimetry for personalized radiopharmaceutical therapy has gained considerable attention. Many methods, tools, and workflows have been developed to estimate absorbed dose (AD). However, standardization is still required to reduce variability of AD estimates across centers. One effort for standardization is the Society of Nuclear Medicine and Molecular Imaging ^177^Lu Dosimetry Challenge, which comprised 5 tasks (T1–T5) designed to assess dose estimate variability associated with the imaging protocol (T1 vs. T2 vs. T3), segmentation (T1 vs. T4), time integration (T4 vs. T5), and dose calculation (T5) steps of the dosimetry workflow. The aim of this work was to assess the overall variability in AD calculations for the different tasks. **Methods:** Anonymized datasets consisting of serial planar and quantitative SPECT/CT scans, organ and lesion contours, and time-integrated activity maps of 2 patients treated with ^177^Lu-DOTATATE were made available globally for participants to perform dosimetry calculations and submit their results in standardized submission spreadsheets. The data were carefully curated for formal mistakes and methodologic errors. General descriptive statistics for ADs were calculated, and statistical analysis was performed to compare the results of different tasks. Variability in ADs was measured using the quartile coefficient of dispersion. **Results:** ADs to organs estimated from planar imaging protocols (T2) were lower by about 60% than those from pure SPECT/CT (T1), and the differences were statistically significant. Importantly, the average differences in dose estimates when at least 1 SPECT/CT acquisition was available (T1, T3, T4, T5) were within ±10%, and the differences with respect to T1 were not statistically significant for most organs and lesions. When serial SPECT/CT images were used, the quartile coefficients of dispersion of ADs for organs and lesions were on average less than 20% and 26%, respectively, for T1; 20% and 18%, respectively, for T4 (segmentations provided); and 10% and 5%, respectively, for T5 (segmentation and time-integrated activity images provided). **Conclusion:** Variability in ADs was reduced as segmentation and time-integration data were provided to participants. Our results suggest that SPECT/CT-based imaging protocols generate more consistent and less variable results than planar imaging methods. Effort at standardizing segmentation and fitting should be made, as this may substantially reduce variability in ADs.

## Visual Abstract

**Figure:**
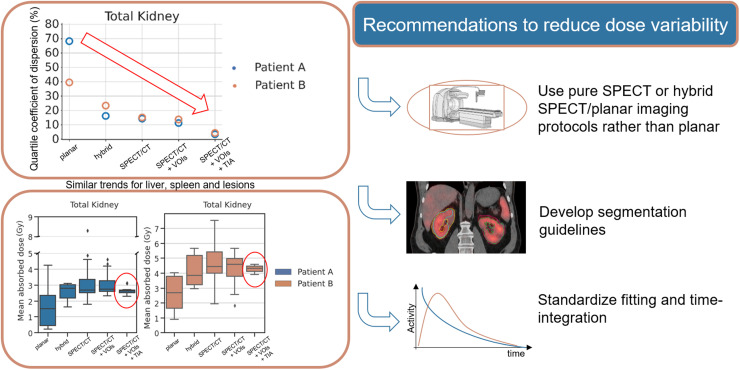

Recent clinical trials have demonstrated favorable patient outcomes and led to U.S. Food and Drug Administration approvals of ^177^Lu-based radiopharmaceuticals for the treatment of neuroendocrine tumors (^177^Lu-DOTATATE (1) in 2018) and metastatic castration-resistant prostate cancer (^177^Lu-PSMA-617 (2) in 2022). These approvals have motivated research for new targets and development of new radiopharmaceuticals by both academia and industry (3–7). Despite the initial promising results for radiopharmaceutical therapies, recurrence has also been reported (8*,*^9^). Patient-specific dosimetry (10) may allow personalization of administered activity to deliver maximized absorbed doses (ADs) to lesions while keeping normal-organ ADs below toxic levels. There is evidence that dosimetry-guided therapy increases the survival of patients who undergo liver radioembolization (11). However, dosimetry calculations are still not routinely performed for radiopharmaceutical therapies, partly because of the lack of standardized dosimetry tools, methods, and protocols. Dosimetry-based therapy-planning approaches are fundamentally limited by the precision of the AD estimates. However, relatively little is known about variability in ADs and the extent to which the different steps of the dosimetry workflow contribute to it.

Dosimetry calculations involve multiple steps (12–14): quantitative imaging of the distribution of the radiopharmaceutical over time, segmentation of lesions and organs of interest, estimation of the total number of disintegrations (time-integrated activity (TIA)) in each target region (e.g., organs and lesions), and conversion of TIA to AD using either organ-level or voxelized dosimetry methods. Alternatively, serial dose-rate images can be calculated first, followed by fitting and integration over time.

To better understand the relative contribution to variability of the various steps of the dosimetry workflow, the Society of Nuclear Medicine and Molecular Imaging (SNMMI) Dosimetry Task Force launched the ^177^Lu Dosimetry Challenge in 2021 (15). The challenge included 5 tasks (T1–T5). Three tasks investigated variability caused by different imaging protocols: serial SPECT/CT (T1), serial planar images (T2), or a hybrid approach (serial planar and 1 SPECT/CT image) (T3). Two additional tasks provided participants with volumes of interest (VOIs) (T4) and TIA maps (T5) with the aim of removing variability in segmentation and integration by removing sources of variability in the serial SPECT/CT workflow. The challenge did not address the impact of variability in ADs caused by image acquisition, calibration, or reconstruction.

The aim of the analysis presented in this work was to assess the source and magnitude of variability in AD estimates, for both organs and lesions, for the different tasks of the ^177^Lu dosimetry challenge and to inform standardization efforts.

## MATERIALS AND METHODS

### Patient Images and Data Collection

**FIGURE 1.**
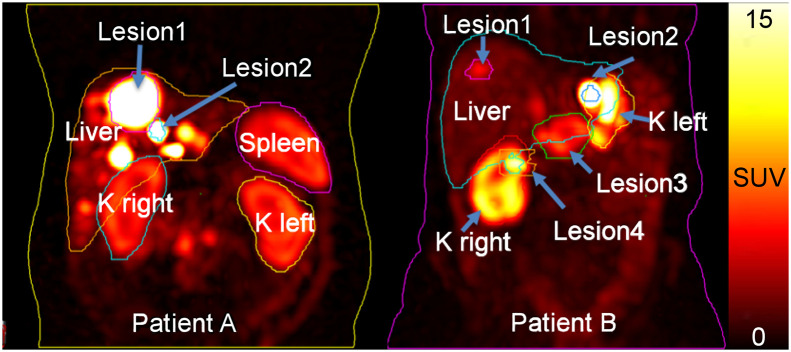
Maximum-intensity-projection images at 24 h after injection of ^177^Lu-DOTATATE for the 2 patients included in the dosimetry challenge. Contours of VOIs provided in T4 and T5 are shown. For patient A, average VOIs as measured from RTstructure files were 1,959 cm^3^ for liver, 247 cm^3^ for spleen, 467 cm^3^ for total kidney, 107 cm^3^ for lesion 1, and 3 cm^3^ for lesion 2; for patient B, they were 1,693 cm^3^ for liver, 229 cm^3^ for total kidney, 11 cm^3^ for lesion 1, 3 cm^3^ for lesion 2, 68 cm^3^ for lesion 3, and 22 cm^3^ for lesion 4. K = kidney.

Datasets of 2 patients who underwent planar and SPECT/CT imaging at 4 time points after administration of ^177^Lu-DOTATATE therapy (16) were shared via the Deep Blue Data repository (https://deepblue.lib.umich.edu/data/collections/hm50ts030?locale=en) (17–21) of the University of Michigan. Sharing of patient images and data was approved by the University of Michigan Institutional Review Board, and both patients gave written informed consent. Table 1 summarizes the data provided. Maximum-intensity-projection images showing the provided VOIs are displayed in Figure 1.

**TABLE 1.** Patient Characteristics, Administered Activities, and Pre- and Post-therapeutic Imaging Information

Patient	Injected activity (GBq)	Diagnostic images	Post-therapy images	Characteristics
A	7.21	Baseline MRI with contrast medium; ^68^Ga PET 185 d before baseline CT and 468 d before first SPECT/CT	SPECT/CT and whole-body planar images on day of treatment and days 1, 4, and 5 after treatment	Two liver lesions were selected for the challenge
B	7.31	Baseline CT with contrast medium; ^68^Ga PET 36 d after baseline CT and 69 d before first SPECT/CT	SPECT/CT and whole-body planar images on day of treatment and days 1, 4, and 8 after treatment	Patient had been splenectomized; 4 lesions were selected for the challenge

Participants reported results on standardized spreadsheets tailored to each task. No lesion AD results were requested in T2 because of the overlap of lesions with organs on planar images. Separate spreadsheets were submitted for each patient. The data requested included information about methods, software used, intermediate values (e.g., VOI volumes, activities, and TIAs), and final ADs. Details of the methodology used for the challenge were given in our previous publication by Uribe et al. (15), including a full list of collected variables. Participants were encouraged to submit multiple calculations for the same patient using different types of software ormethodology (e.g., organ-based vs. voxel-based calculations). Participants were asked to briefly describe their dosimetry workflow in addition to the spreadsheet; unfortunately, however, only 1 participant mentioned partial-volume correction.

### Data Collation

Data from all received submissions were extracted using the Python data analysis library (Pandas, version 1.3.5) and Python (version 3.9.5). All data were concatenated into a single data frame with columns corresponding to the specific variables collected. A full glossary of variables and column descriptions can be found in Supplemental Tables 1 and 2 (supplemental materials are available at http://jnm.snmjournals.org), as well as in the GitHub repository of the ^177^Lu Dosimetry Challenge (https://github.com/carluri/snmmi_dosimetry_challenge).

We curated the data, including identifying typographical, orientation (left/right ambiguity), and unit conversion issues and errors and evaluating data completeness. Participants were contacted for confirmation and clarification as needed. Results that were identified as containing mistakes in the calculations were removed from the analysis. A detailed description of the data curation process can be found in the supplemental material.

### Statistical Analysis

Descriptive statistics, such as quartiles, means, and standard deviations (SDs) of ADs, were calculated separately for each task, patient, organ, and lesion. The quartile coefficient of dispersion (QCD) was calculated as the ratio of the difference between the 75th and 25th quartiles and the sum of the 75th and 25th quartiles of the data. The QCD was chosen since it is less sensitive to outliers in the data than is the coefficient of variation. For a normal distribution, the coefficient of variation is 1.4826 times the QCD.

A mixed-effects model was used to compare ADs among T1, T2, and T3 and, separately, among T1, T4, and T5. The model included the task as a fixed effect and the participant as a random effect. The analysis was performed separately for each patient and organ or lesion. Restricted maximum likelihood was applied to estimate the parameters in the models. All tests were 2-sided, and *P* values of 0.05 or less were considered to indicate statistically significance differences. The analysis was performed with Python (version 3.9.5) and R (version 4.1.0).

## RESULTS

### General Observations

We received 321 submissions from 51 institutions (Asia, 8; Australia, 2; Europe, 18; North America, 22; and South America, 1). Table 2 summarizes the submissions per task and patient. Some participants submitted multiple spreadsheets using different dosimetry methods (e.g. organ-level and voxelized).

**TABLE 2.** Number of Submissions per Task and per Patient

Patient	Submissions (*n*)				
	T1	T2	T3	T4	T5
A	63	13	16	40	31
B	63	11	14	40	30

16 participants (institutions) submitted all tasks, and 10 submitted T1, T4, and T5.

Both open-source and commercial dosimetry softwares were used, with details provided in Supplemental Figures 1 and 2. Approximately 27% of submissions used an in-house dose calculation approach.

### Absorbed Doses

**FIGURE 2.**
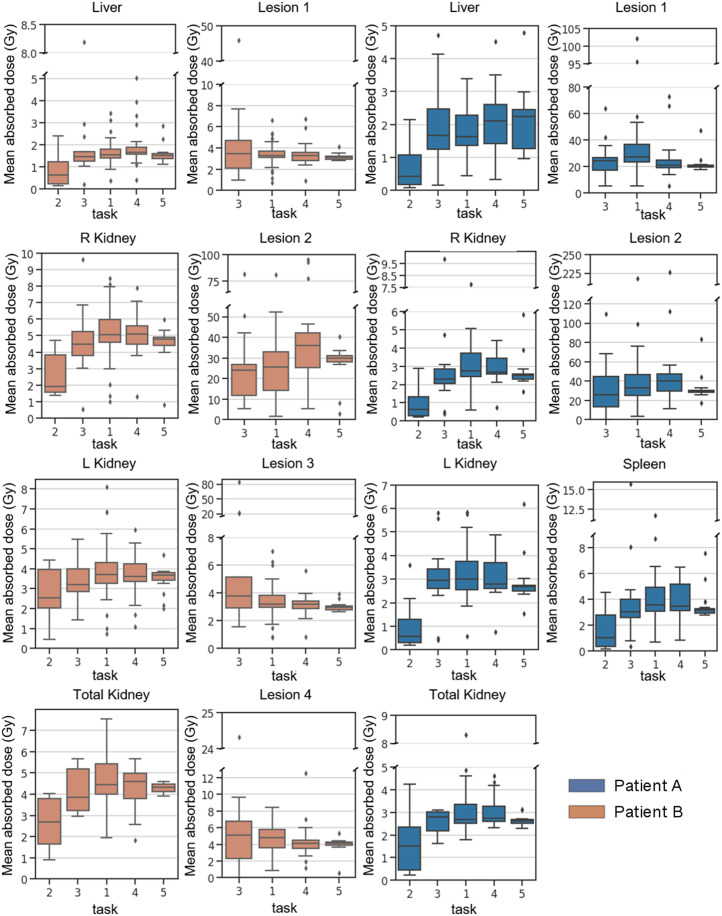
Mean organ and lesion AD in Gray per task and per patient. Patient B had been splenectomized. T2 was based on planar images, T3 used hybrid imaging protocol of multiple planar images and 1 SPECT/CT image, T1 used multiple SPECT/CT images, T4 used multiple SPECT/CT images and provided VOIs, and T5 was based on provided TIA image and VOIs.

Figure 2 shows the distribution of mean ADs in organs and lesions from all submissions (after data curation). The numeric values of the descriptive statistics underlying Figure 2 can be found in Supplemental Table 1.

Table 3 shows the percentage difference calculated as the difference between the median of all submissions per task taking the median of all submissions from T1 as a reference, as well as the median of all submissions from T4 and T5 as a reference. Percentage differences were averaged separately for patients and for organs and lesions.

**TABLE 3.** Percentage Difference Between Medians of All Submissions per Task Relative to T1 and T4 Relative to T5

Patient	Organ	T2 vs. T1	T3 vs. T1	T4 vs. T1	T5 vs. T1	T4 vs. T5
A	Liver	−74%	2%	29%	38%	−6%
	Spleen	−71%	−15%	−2%	−12%	11%
	R kidney	−77%	−17%	−3%	−9%	7%
	L kidney	−81%	−2%	−7%	−11%	4%
	Total kidney	−43%	4%	3%	−2%	5%
	Lesion 1		−10%	−23%	−25%	3%
	Lesion 2		−23%	21%	−10%	34%
B	Liver	−60%	−6%	6%	−3%	9%
	R kidney	−62%	−11%	1%	−5%	6%
	L kidney	−31%	−14%	−3%	−1%	−2%
	Total kidney	−40%	−13%	4%	−2%	6%
	Lesion 1		6%	−1%	−5%	5%
	Lesion 2		−6%	40%	16%	20%
	Lesion 3		19%	0%	−7%	7%
	Lesion 4		6%	−14%	−16%	1%

In general, ADs calculated from planar imaging protocols (T2) were lower than pure SPECT or hybrid protocols (Fig. 2). ADs for organs from T2 underestimated those from the pure SPECT protocol of T1 on average by 60%, ranging from −81% to −31% (Table 3). In contrast, the ADs of the hybrid protocol (T3) were similar to those of the pure SPECT/CT protocol (T1) for organs (on average 8% lower, ranging from −17% to 4%, Table 3). On average, the ADs for all organs were within ±10% of one another for T1, T4, and T5 (Table 3), suggesting no substantial bias between SPECT-based tasks. For lesions, a larger spread of values was observed (Fig. 2), but average percentage differences were within ±12%, ranging from −25% to +40% (Table 3). The provision of TIA maps in T5 yielded slightly smaller ranges in percentage difference of the medians of organ and lesion doses of T4 relative to T5 (Table 3).

### Quantification of Variability

**FIGURE 3.**
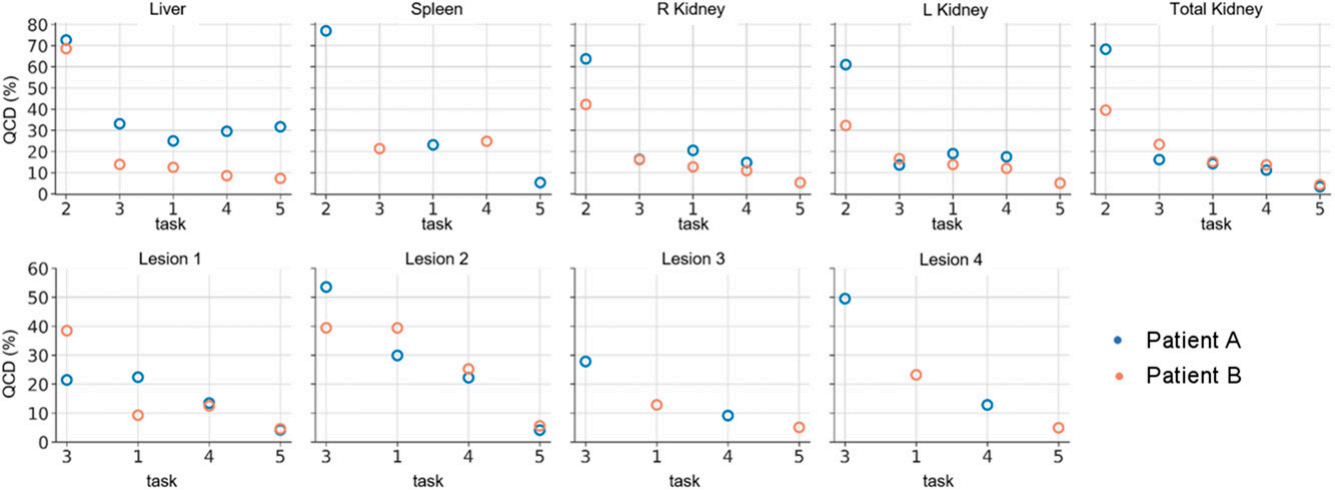
QCD per task, organ, and patient.

Figure 3 and Table 4 show the QCDs of ADs for all tasks averaged over organs and lesions. QCDs per organ and patient are provided in Supplemental Table 1. The QCDs for T2 were large, with an average of 69% for patient A and 46% for patient B (Table 4). The hybrid (T3) and pure SPECT (T1) protocols had similar QCDs for organs: on average 20% for patient A and 17% and 14% for patient B. Overall, the variability was reduced as segmentation and TIA activity data were provided (i.e., T1 to T4 to T5) (Figs. 2 and 3; Table 4). For the pure SPECT protocols (T1, T4, and T5), there was an overall reduction by a factor of about 1.5 in lesion QCD when VOIs were given to participants (T1 vs. T4, Table 4); the change for organs was smaller. Larger QCDs were observed for the smaller lesions (lesion 2 of patient A and lesion 2 of patient B) in T1, when segmentation and time integration were performed by participants. There was a substantial reduction in QCD for both organs and lesions when both VOIs and TIA maps were given to participants (T5, Table 4), resulting in QCDs of less than 7% for organs (excluding the liver of patient A) and less than 6% for lesions, independent of lesion size.

**TABLE 4.** QCD per Task, Organ, and Patient

Organ	Patient A					Patient B				
	T2	T3	T1	T4	T5	T2	T3	T1	T4	T5
Liver	73%	33%	25%	30%	32%	69%	14%	13%	9%	7%
Spleen	77%	21%	23%	25%	5%					
R kidney	64%	16%	21%	15%	5%	42%	16%	13%	11%	5%
L kidney	61%	14%	19%	18%	5%	32%	17%	14%	12%	5%
Total kidney	68%	16%	14%	11%	3%	40%	23%	15%	14%	4%
Lesion 1		21%	22%	13%	4%		38%	9%	13%	5%
Lesion 2		54%	30%	22%	4%		39%	39%	25%	6%
Lesion 3							28%	13%	9%	5%
Lesion 4							50%	23%	13%	5%

### Statistical Analysis

The results of the statistical comparisons between ADs calculated for the different tasks are given in Table 5. In general, statistically significant differences were observed between T1 (pure SPECT) and T2 (planar imaging) but not between tasks that involved a SPECT scan, although there were a few exceptions.

**TABLE 5.** *P* Values for Comparisons of ADs Between Various Tasks and T1

Organ	Planar-, hybrid-, and SPECT-based tasks				Purely SPECT-based tasks			
	T1 vs. T2		T1 vs. T3		T1 vs. T4		T1 vs. T5	
	Patient A	Patient B	Patient A	Patient B	Patient A	Patient B	Patient A	Patient B
Liver	<0.01*	0.03*	0.09	0.08	<0.01*	0.01*	<0.01*	0.89
Spleen	<0.01*		0.87		0.63		0.12	
R kidney	<0.01*	0.01*	0.75	0.61	0.65	0.84	0.14	0.07
L kidney	<0.01*	0.13	0.87	0.67	0.86	0.44	0.18	0.22
Total kidney	0.01*	0.15	0.31	0.88	0.59	0.22	0.27	0.73
Lesion 1			0.15	0.02*	0.02*	0.92	<0.01*	0.40
Lesion 2			0.82	0.74	0.08	<0.01*	0.63	0.24
Lesion 3				<0.01*		0.12		0.02*
Lesion 4				0.21		0.14		0.06

* Statistically significant difference.

## DISCUSSION

All of the different steps in the dosimetry workflow potentially contribute to variability in AD estimates. First, the choice of imaging protocol (i.e., planar, SPECT, or hybrid imaging) can affect the measurement of the activity estimates that are the basis for dosimetry calculations. Second, the segmentation of organs and lesions can also affect the ADs. For voxel-based dosimetry, segmentation defines the spatial extent of VOIs where the AD is averaged, whereas for organ-based dosimetry it defines the organ mass and activity. The effect of segmentation on the AD is complicated since both the numerator (energy) and the denominator (mass) in the definition of dose (i.e., joules per kilogram) are affected. The estimation of total number of decays performed by curve fitting and time integration is influenced by the choice of fit function and the temporal limits of integration, neither of which is currently standardized. Finally, the choice of dosimetry method, software, and source of S-values or dose kernel can affect the final AD. The SNMMI ^177^Lu Dosimetry Challenge was designed to assess the variability in ADs caused by imaging protocol (T1, T2, and T3), segmentation (T4), and time-integration and dosimetry method (T5) on the final dosimetry results.

The planar protocol (T2) resulted in lower ADs by a factor of 2 and higher average QCDs than SPECT-based protocols, and these differences were statistically significant (Table 5). Lesion doses were not requested for T2 (planar protocols) because of the overlap with organs. Of note, T2 had the smallest number of submissions, with only 13 and 11 submissions for patients A and B, respectively.

The differences between ADs from the hybrid (T3) and pure SPECT (T1, T4, and T5) protocols were generally not statistically significant (Table 5). With the hybrid approach, the issues with overlapping structures are substantially reduced by use of the SPECT/CT image but can still affect the shape of the time–activity curve, especially for objects in high-uptake regions such as the lesions (Fig. 1). These results are consistent with previous reported data about the accuracy and precision of SPECT and hybrid protocols as compared with planar protocols (22–26).

The differences in ADs for the purely SPECT-based T1, T4, and T5 were generally small (Fig. 2; Table 3) and were not statistically significant for most organs and lesions (Table 5). However, statistically significant differences for the liver were observed, as can be explained by the presence of lesions in the livers of both patients (Fig. 1)—lesions might not have been excluded in the segmentation of healthy liver by all participants. This possibility suggests that standardization of segmentation methodologies should be considered to reduce variability. As demonstrated in Figure 2, the ranges of dose results were reduced when VOIs and TIA maps were provided, that is, comparing T1 with T4 and T5.

The largest average QCDs were found for the planar protocol, that is, T2 (Fig. 3; Table 4). We observed larger QCDs for the smaller lesions (Fig. 3, lesion 2 of patients A and B) than for the larger lesions. This result is expected given the difficulty and subjectivity associated with lesion segmentation and mass definition for those structures. This difficulty can further be related to the partial-volume effect, which is more pronounced for smaller lesions such as lesion 2 of patients A and B. In general, the QCDs, reflecting variation in ADs, were reduced as more information was provided to participants (i.e., T1 to T4 to T5), even for the challenging small lesions. Average organ QCDs changed little when VOIs were provided to participants (T1 to T4, Table 4), whereas lesion QCDs decreased by a factor of approximately 1.5. The QCDs of T5 were as low as 10% and 5% on average for all organs and 4% and 5% for all lesions of patients A and B, respectively (Table 4). The vast decrease in QCD between T1 and T5 from 30% to 4% for the small lesion 2 (∼3 mL) of patient A and from 39% to 6% for lesion 2 (∼3 mL) of patient B, compared with the moderate QCD decrease from 22% to 4% for lesion 1 (∼107 mL) of patient A and from 13% to 5% for lesion 3 (∼68 mL) of patient B, indicates that both segmentation and integration represent large sources of variability, especially for smaller objects. Segmentation can be further complicated when lesions are within an organ with little difference in contrast. The largest organ QCDs from all submissions for T5 were 32% and 7% for the liver of patients A and B, respectively (Fig. 3; Table 4; Supplemental Table 1). The size of this variation was unexpected given the data provided to the participants in T5. We attribute this variation to the presence of liver lesions and different decisions made by participants about what to include in the liver VOI (i.e., removing all lesions or only the lesions indicated by the challenge, Fig. 1). In general, the segmentation and TIA data provided in T4 and T5 substantially reduced variability as assessed by QCD (Fig. 3) and with respect to Figure 2, strongly suggesting that efforts to standardize segmentation (e.g., whether to include suspected lesions in normal tissues and whether to include the medulla and pelvis in kidney VOIs) may substantially reduce variability. Furthermore, providing TIA maps (i.e., standardization of fitting and integration) strongly reduced the variability in ADs and points to the integration approach as a source of substantial variability and a target for standardization.

The remaining variability in T5 can be attributed to several sources. Since this step included solely the conversion from TIA to AD, it may be related to differences in S values, dose kernels, or Monte Carlo simulations. Generally, these differences have been found to be small (<5%) (27–29), as is consistent with our independent findings for this dataset (30). Another potential source of variability is application of mass scaling to the S values (31) or density weighting to the dose kernels (27), both of which options are usually available in dosimetry software. Future analysis will focus on the effect of these factors on T5.

A limitation of this work is that it is based on only 2 patient datasets; this number was selected as a compromise between gaining more information on interpatient variability and the desire to attract a larger number of voluntary participants. The 2 patients chosen, however, illustrate some important characteristics and common challenges in the dosimetry workflow related to imaging protocol (planar vs. SPECT), segmentation, and integration. Specific characteristics of interest included a large tumor burden in the liver, significant differences between right and left kidney volumes, significant differences from standard phantom organ volumes, and lesion size and proximity to other high-uptake structures. In addition, some unintentional sources of variability are inherent in the design and implementation of the SNMMI ^177^Lu Dosimetry Challenge. For example, VOIs were provided both in the radiotherapy structure set (RTSTRUCT) of the DICOM standard and as voxelized masks to accommodate different capabilities in software available to participants. The process of voxelizing the RTSTRUCT resulted in different volume and activity estimates between the contours and the masks depending on the software used for the analysis. For example, one software package used by the organizers allowed the contours to include subvoxels, but the masks always contained complete voxels. Generally, the contour interpolation into subvoxels should be disabled for dosimetry purposes. This disabling resulted in average differences in volumes of 8% (range, 4%–14%) for organs and of 20% (range, 8%–37%) for lesions. Thus, the use of RTstructure by some participants and masks by others added to the variability in ADs. Similarly, VOIs were provided at each imaging time point, whereas the TIA map for T5 was generated using the first imaging time point as a reference. Applying to the TIA map the VOIs from a time point other than the first will consequently lead to different results. Although reflective of differences that may be observed at different sites in a clinical environment, the magnitude of the contribution of these sources to overall variability is likely specific to the design and implementation of the challenge. We did not expect that the provision of VOIs in RTstructure and mask format at multiple time points would affect the ADs and thus did not act to minimize this source of variability. Nevertheless, this difference when saving VOIs does highlight the potential for variability due to differences in software implementations and settings. Finally, this challenge did not address the impact of image acquisition, reconstruction, and quantification, which are also considered to be major contributors to variability of dose estimates.

The analysis of the SNMMI ^177^Lu Dosimetry Challenge data on overall dose variability elucidated several areas in which standardization or harmonization may be important to reduce variability across sites and methods. Our initial recommendations to reduce the variability of dose calculations based on the results of our analysis are as follows.

First, pure SPECT or hybrid SPECT/planar imaging protocols should be used for dosimetry instead of planar imaging–only protocols. The results presented here indicate that this recommendation would reduce variability and suggest a reduced bias.

Second, the development of segmentation guidelines for organs and lesions can help standardize the process and reduce the variability observed in this study. For example, guidance can be issued on how to deal with overlapping regions such as lesions in the liver and which regions of the kidney such as the renal pelvis should be included in the segmentation. In the meantime, we believe that publications should explicitly detail how these procedures are being performed to ensure that a correct comparison between results is being made.

Third, standardizing the use of fitting functions and integration methods would achieve some meaningful reduction in dose variability based on the reduction in variability from T4 to T5.

Fourth, more detailed and standardized reporting (32*,*^33^) of such details as dosimetry method (voxelized vs. organ-based), software used, appropriate use of mass scaling, and user decisions regarding the inclusion of kidney substructures should be developed to enable comparison of results from different centers and in different trials. A standardized nomenclature as emphasized in MIRD pamphlet 21 (34) would facilitate this reporting.

## CONCLUSION

This analysis of all tasks of the SNMMI ^177^Lu Dosimetry Challenge highlights the need to move toward SPECT-based imaging protocols for dosimetry of radiopharmaceutical therapies. Standardizing segmentation and fitting methods and decisions is essential to reducing variability in AD. Removing these sources of variation from the dosimetry workflow reduced the variability to below 10% for organs and lesions.

## DISCLOSURE

This work was partly supported by the SNMMI Value Initiative. Yuni Dewaraja acknowledges funding from grant R01CA240706 awarded by the National Cancer Institute for patient studies and resources made available by the University of Michigan Deep Blue Data Repository for data sharing. Eric Frey is a cofounder and part-owner of Rapid, LLC; receives royalty income from GE Healthcare; and acknowledges support from grants R44CA213782 and R01CA240779 awarded by the National Cancer Institute. Carlos Uribe acknowledges funding from Natural Science and Engineer Research Council (NSERC) discovery grant RGPIN-2021-02965. No potential conflict of interest relevant to this article was reported.

## Notes

KEY POINTS **QUESTION:** What is the overall variability in AD estimates in radiopharmaceutical therapy calculated using different methods at different centers? **PERTINENT FINDINGS:** Planar imaging protocols resulted in substantially lower estimates of AD and the largest variability when compared with protocols that used SPECT/CT images. Overall variability using serial SPECT/CT as measured by QCD was less than 26% for organs and lesions. The results suggest that standardization of integration and segmentation may further reduce variability. **IMPLICATIONS FOR PATIENT CARE:** Standardization of the dosimetry workflow will reduce variability in AD estimates and ultimately improve reliability—essential for dosimetry-based personalization of radiopharmaceutical therapies.
